# Vulcanization Characteristics and Static/Dynamic Mechanical Properties of Chlorinated Butyl Rubber Matrix Materials

**DOI:** 10.3390/polym17060708

**Published:** 2025-03-07

**Authors:** Kai Wang, Hengxu Lv, Zhixin Liu

**Affiliations:** China Automotive Technology and Research Center Co., Ltd., Tianjin 300300, China; wangkai2021@catarc.ac.cn (K.W.);

**Keywords:** chlorinated butyl rubber, natural rubber, ethylene propylene diene monomer rubber, damping performance, vulcanization properties

## Abstract

The damping performance of chlorinated butyl rubber (CIIR) is exceptional; however, its poor processability during vulcanization can lead to numerous defects. Natural rubber (NR) and ethylene propylene diene monomer rubber (EPDM) were selected to blend with CIIR for improving its processing performance. Their effects on the vulcanization characteristics, mechanical properties, and damping performance were investigated. Blending CIIR with NR can considerably increase the vulcanization speed of the rubber compound and improve production efficiency. The tensile strength of the vulcanizate first increases with an increase in the dosage of NR in NR/CIIR, and subsequently, it decreases before increasing again. The tensile strength first increases and then decreases with an increase in the EPDM dosage in EPDM/CIIR vulcanizate. The tensile strength increases by 15.6%when the EPDM dosage is 60 and 80 phr. EPDM and NR have similar effects on the damping performance of CIIR, which were evaluated by fitting the data of loss factor (∆*tanδ*) versus NR or EPDM dosage. Therefore, the quantity of NR or EPDM can be conveniently calculated based on performance requirements when designing the formula of the CIIR matrix materials.

## 1. Introduction

Butyl rubber (IIR) [1] is a linear polymer prepared by the cationic polymerization of isobutylene with a small quantity of isoprene. Research efforts have focused on producing halogenated IIRs, such as CIIR [2], to improve compatibility between IIR and unsaturated rubber and strengthen self and mutual adhesion. Chlorination predominantly occurs in the isoprene segment and the main chain structure of IIR remains unchanged, enabling CIIR to inherit the advantages of IIR, such as heat resistance, ozone resistance, and good airtightness [3], while demonstrating enhanced compatibility and adhesion [4]. Given that each isobutylene molecule contains two methyl groups, the CIIR molecular chain formed after polymerization comprises many side methyl groups, which enable intense friction, large hysteresis loss [5], and an evident damping effect [6] when relative motion occurs between different molecular chains. The damping performance of CIIR is exceptional among all synthetic rubbers [7,8,9,10].

NR is a natural polymer compound with cis-1,4-polyisoprene as the main component, 91% to 94% of which is rubber hydrocarbon (cis-1,4-polyisoprene), and the rest are non-rubberized substances such as proteins, fatty acids, ash, and sugars. EPDM is a copolymer of ethylene, propylene and a small amount of non-conjugated diolefin, which is a kind of ethylene propylene rubber, because its main chain is composed of chemically stable saturated hydrocarbons, and only contains unsaturated double bonds in the side chain.

Based on our previous study, CIIR has poor processability without the use of excessive fillers because internal bubbles cannot be discharged effectively during vulcanization, thereby leading to a large number of defects. NR [11] and EPDM [12,13] are selected as the blending rubber of CIIR [14,15] to improve the processing performance of CIIR. Their effects on the vulcanization characteristics, mechanical properties, and damping performance of CIIR were investigated.

Ma et al. [16] used dicumyl peroxide (DCP) to in situ enhance the compatibility of poly-β-hydroxybutyric (PHB)(V)/Polybutylene succinate (PBS) mixtures. The decomposition of DCP generates free radicals that trigger the reaction between PHB (V) and PBS, thereby resulting in the formation of PHB (V)—g-PBS copolymers, partially crosslinking the network structure in the mixture and enhancing the interfacial bonding strength between the two phases, which significantly improves the mechanical properties of the blend material.

Abtahi [17] studied the effect of the crosslinking agent triallyl cyanate (TAC) on the vulcanization and mechanical properties of SiO_2_/EPDM and found that TAC can improve the interaction between EPDM and SiO_2_, increase the apparent crosslinking density, and enhance the mechanical properties of the material.

Fu [18] used benzoyl peroxide as an initiator to prepare EPDM-g-MMA St by the solution graft copolymerization of methyl methacrylate (MMA) and styrene (St) onto EPDM in the toluene/n-hexane solvent. An EPDM-g-MMA St/MS resin blend (MES) was prepared by melt blending and found that the addition of the graft polymer improved the toughness of the resin. Kim [19] prepared a graft polymer EPDM-g-CCA by the free radical melt polymerization of EPDM and citric acid (CCA). Then, the graft polymer was fused with various amino acids to form amide copolymers (EPDM-g-CCA2-Am, EPDM-g-CCA-7-Am, and EPDM-g-CCA-12-Am, where n represents the carbon number of the amino acid). Then, EPDM-g-CCA/n-Ams was fused with zinc oxide/zinc stearate to prepare ionomers (EPDM-g-CCA/n-Am/Io). Comparative studies revealed mechanical properties, compression set resistance, and recyclability of EPDM-g-CCA/n-Am when n did not exceed 7; these values were superior to those of the EPDM-g-CCA ionomer thin film samples. Botros [20] synthesized the MAH-g-EDM by grafting maleic anhydride (MAH) and EPDM in a double roll open mill. The graft copolymer was used as a compatibilizer for EPDM/NBR compatibilization and blending, which revealed that the addition of the graft polymer improved the mechanical properties, heat resistance, and UV stability of the blended system.

In addition, nanofillers gained considerable research interest in improving the compatibility of immiscible mixtures [21,22,23]. The incorporation of nanoparticles has become an economical solution because of its simplicity and effectiveness. Researchers such as Lipatov and Nesteroy [24] and Ginzburg [25] explored the physical mechanisms of nanofiller-induced compatibility and morphology control in phase separated blends. Compatibility arises from the preferential positioning of nano fillers, reducing interfacial tension, inhibiting agglomeration, and improving morphology. Furthermore, morphological refinement occurs through two mechanisms depending on the localization of nanoparticles. Nanofillers can delay relaxation kinetics when selectively incorporated into a polymer phase [26], thereby preserving finer structures. Nanofillers can form a filtration network when they are separated into a continuous phase, thereby increasing viscosity and dynamically capturing finely dispersed domains, preventing coarsening [27,28,29]. Alternatively, nanoparticles can promote finer morphology and inhibit agglomeration through steric hindrance effects [30] when located at the interface between immiscible biopolymer phases. Their high surface area and ability to cover interfaces provide coupling interactions between polymers [31].

In our research on the processing and application of CIIR, we discovered that CIIR exhibits poor processability. Due to its excellent airtightness, without the extensive use of fillers, internal bubbles cannot be effectively discharged during vulcanization, resulting in numerous defects. Rubber blending is one of the most important and effective means of rubber modification. To improve the processability of CIIR, we selected NR and EPDM as blends with CIIR, and explored their effects on the vulcanization properties, mechanical properties, and damping properties of CIIR.

## 2. Materials and Methods

### 2.1. Materials

Ethylene Propylene Diene Monomer (EPDM) used in this work was provided by Mitsui Chemical, Tokyo, Japan, and the ENB and ethylene contents were 4.1 wt% and 48 wt%, respectively. Chlorinated Butyl rubber (CIIR) with chlorine and isoprene contents of 1.25 wt% and 2.0 mol%, respectively, was purchased from Exxon Mobil Corporation, Spring, TX, USA. Natural rubber (NR) was 3#Bacon rubber supplied by Thailand. Zinc oxide and Sulfur were provided by Rhein Chemie GmbH, Mannheim, Germany. Carbon black (N550) with the particle size of ca. 50 nm was supplied by Cabot Co., Ltd., Boston, MA, USA. The curing system consisting of sulfur and 2,2′-dibenzothiazoledisulfde (DM), and activa-tion system including stearic acid and zinc oxide were offered by Rhein Chemie Co., Ltd. Paraffin oil with the density of 0.899 g/cm^3^ (15 °C) and the relative molecular weight of 720, and N-1,3-dimethylbutyl-N′-phenyl-p-phenylenediamine (antioxidant 4020) were purchased from commercial sources.

### 2.2. Experimental Formula

The experimental formulation used in this study is shown in Table 1 and Table 2.

### 2.3. Blends Preparation

According to the formula given in Table 1 and Table 2, NR/CIIR and EPDM/CIIR raw rubbers were blended firstly in an internal mixer (Shanghai KCCK Co., Ltd., XSM-500, Shanghai, China) for 1 min, then the zinc oxide, stearic acid and antioxidant were added and mixed further for 1 min. At last, the carbon black and paraffin oil were added and mixed for 4 min to obtain the masterbatch. The mixing temperature was set at 55 °C, and the rotor speed was set to 60 rpm. The obtained masterbatch was mixed with sulfur and DM by a two-roll mill (BOLON Precision Testing Machines Co., Ltd., BL-6175, Dongguan, China) with a roller speed ratio of 1:1.2 in order to obtain the compounds. After being kept at room temperature for 12 h, the compounds were vulcanized on a hydraulic press at 170 °C and t_90_ + 5 min as curing time determined by the Moving Die Rheometer.

### 2.4. Characterization Methods

A moving die rheometer (Alpha Technology, MDR2000, Beirut America) was used to test the vulcanization curve of rubber at 170 °C according to GB/T16584-1996 [32]. The tensile properties test was conducted using an electronic tensile machine (Zwick Z030, Ulm, Germany) in accordance with GB/T 528-2009 [33]. The test was performed at room temperature with a tensile rate of 200 mm/min. A dynamic mechanics analyzer (HITACHI, NEXTA DMA200, Tokyo, Japan) was used to test the energy loss factor and the storage modulus.

The steps to obtain ∆*tanδ* are as follows:

(1) Frequency selection: To cover a broad frequency range, four frequencies (1 Hz, 10 Hz, 39.8 Hz, and 100 Hz) were selected.
(2) ∆*tanδ* calculation: At the selected frequency, ∆*tanδ* between blends with different NR contents and NR0 were calculated.
(3) Step (2) was repeated to obtain ∆*tanδ* at different frequencies.
(4) ∆*tanδ* was fitted against NR content.

## 3. Results and Discussion

### 3.1. Vulcanization Characteristics of EPDM/CIIR Blends and NR/CIIR Blends

**Figure 1:**
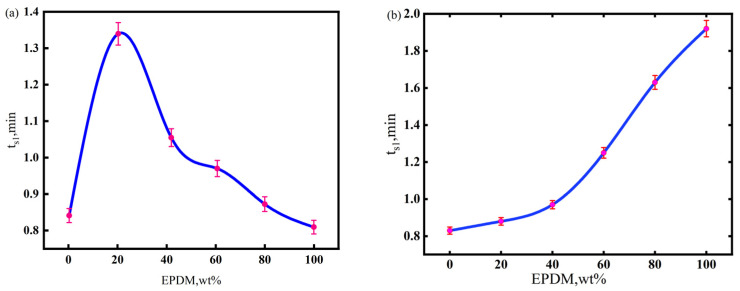
Scorch time of NR/CIIR compound (**a**) and EPDM/CIIR compound (**b**).

The processibility of the CIIR matrix materials was assessed based on their scorch time(ts1) [34], which refers to the time for the torque value to increase by (MH-ML) × 10% after the rubber material reaches the minimum torque ML in the mold cavity, where MH denotes the sum of chemical crosslinking and the physical entanglement of the vulcanizate, and ML indicates viscosity of rubber before vulcanization. The scorch times (denoted as ts1) of NR/CIIR and EPDM/CIIR compounds are shown in Figure 1. A longer scorch time corresponds to better processability.

The scorch time initially increases and then decreases with an increase in the NR component in the matrix [35]. The scorch time of the rubber compound is the longest when the NR dosage is 20 phr (the total mass of raw rubber is regarded as 80 phr), which suggests the best processability of NR20. Macroscopic features on the vulcanization curve can closely correlate with microscopic reactions during rubber vulcanization. In a sulfur vulcanization system containing accelerators, the induction period refers to the concentration accumulation stage when rubber macromolecules with polysulfide accelerator pendant groups (vulcanization precursors) are generated. Vulcanization starts once the precursor concentration reaches a threshold. Most NR molecular chains are first converted to precursors at the vulcanization temperature (170 °C) when 20 phr of NR is used in NR/CIIR. However, the vulcanization reaction is not initiated until a sufficient quantity of precursors is generated in the CIIR phase. When the NR dosage increases from 40 to 80 phr, the variation in ts1 (the time when the torque reaches 10% of the maximum torque) can be explained by the required concentration to initiate the vulcanization reaction. However, the concentration of the vulcanization precursor formed by the NR macromolecules at this time is sufficient to trigger the vulcanization reaction, thereby resulting in a continuous decrease in ts1.

The scorch time of the rubber compound is prolonged by increasing the EPDM content in the matrix. Furthermore, the scorch time becomes significantly longer when the EPDM dose exceeds 40 phr because the main chain of the EPDM molecule is saturated, with limited numbers of active sites on the side chains. Although the carbon chain of CIIR is similar to that of the EPDM, the introduced chlorine atoms activate double bonds, shortening the scorch time.

**Figure 2:**
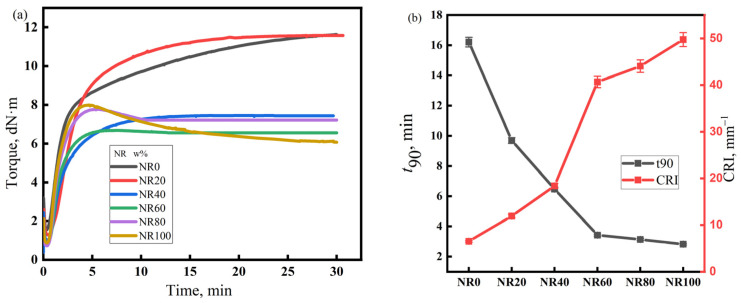
Vulcanization characteristics of the NR/CIIR compound, (**a**) Torque of the NR/CIIR compound, (**b**) t_90_ of the NR/CIIR compound.

The vulcanization characteristics of the rubber compounds with different NR/CIIR ratios are shown in Figure 2. The left panel of Figure 2 depicts the torque versus time curves during the vulcanization process of rubber compounds with different NR/CIIR ratios, while the right panel shows the t_90_ and CRI values (unit: min^−1^) derived from these vulcanization curves for each rubber compound, where t_90_ represents the time when the torque reaches 90% of the maximum torque, and the cure rate index (CRI) represents the speed of rubber vulcanization reaction. The calculation equation for CRI is CRI = 100/(t_90_ − t10). Under the current vulcanization conditions, NR80 and NR100 show a vulcanization reversion phenomenon, which reduces the strength of NR/CIIR. The vulcanization flatness of the rubber compound is excellent when the NR content is less than 80 phr. Therefore, when blending NR and CIIR, an appropriate amount of NR can prevent vulcanization reversion and improve the strength of NR/CIIR vulcanizates. The t_90_ of the rubber compound decreases rapidly with an increase in the NR content. When the dosage of NR is 20 phr, t_90_ decreases by 40.2%, and CRI increases by 132%, thereby demonstrating that blending CIIR with NR can greatly increase the vulcanization speed of the rubber compound and improve production efficiency [36].

**Figure 3:**
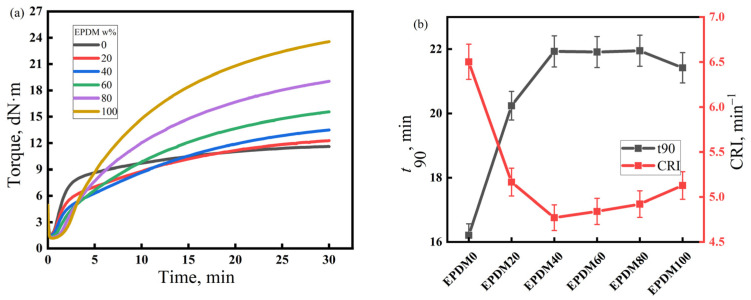
Vulcanization characteristics of EPDM/CIIR compound, (**a**) Torque of the EPDM/CIIR compound, (**b**) t_90_ of the EPDM/CIIR compound.

The vulcanization characteristics of the rubber compounds with different EPDM/CIIR ratios are shown in Figure 3. Similarly to Figure 2, the left and right subplots of Figure 3, respectively, depict the vulcanization curves of rubber compounds with different EPDM/CIIR ratios, as well as the values of t_90_ and CRI. The effects of EPDM dosage on t_90_ and the CRI of the EPDM/CIIR compounds are similar to that on the scorch time, i.e., the vulcanization speed of the rubber compound slows down with an increase in the EPDM dosage. However, the effect eventually diminishes with an increase in the dosage of EPDM. When 20 phr EPDM is blended, t_90_ increases by 24.9%, and CRI decreases by 20.6%.

ML represents the minimum torque, reflecting the flowability of the mixed rubber in the mold cavity; MH represents the maximum torque and is a measure of the shear modulus or hardness of the adhesive material. The difference between the two reflects the crosslinking density (the larger difference, the higher crosslinking).

**Figure 4:**
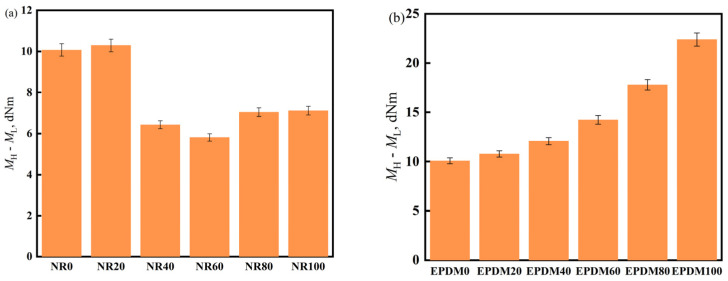
M_H_-M_L_ of NR/CIIR vulcanized rubber (**a**) and EPDM/CIIR vulcanized rubber (**b**).

In Figure 4a, it can be seen that when the NR content is 20%, the difference between the highest and lowest torques is the largest, indicating the highest crosslinking density. When the NR content is 60%, the difference decreases by nearly 40%; in Figure 4b, the maximum torque of the vulcanizate increases with an increase in EPDM dosage, whereas ML remains unchanged, suggesting that EPDM can increase the crosslinking density of the EPDM/CIIR vulcanizate.

### 3.2. Quasi-Static Mechanical Properties of EPDM/CIIR Blends and NR/CIIR Blends

**Figure 5:**
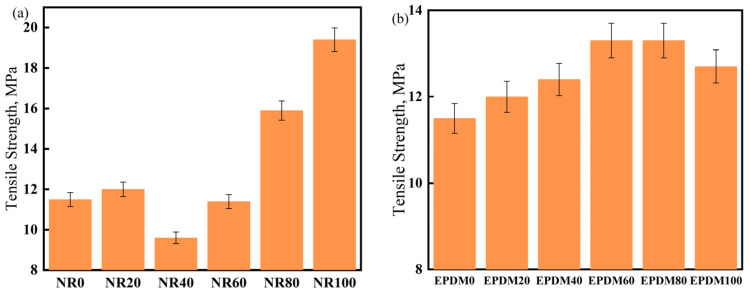
Tensile strength of NR/CIIR vulcanized rubber (**a**) and EPDM/CIIR vulcanized rubber (**b**).

Most damping materials are fixed to structural materials for mitigating vibrations. However, a few applications such as shock-absorbing bearings have certain strength requirements for the damping material. Therefore, it is necessary to study the tensile strength of the CIIR matrix materials. The effect of the blending dosages of NR or EPDM on the tensile strength of NR/CIIR or EPDM/CIIR vulcanizates is shown in Figure 5.

With an increase in the NR content, the tensile strength of the vulcanizate increases first, then decreases, and then increases again. Compared to NR0, the tensile strength of the vulcanizate increases slightly at an NR dosage of 20 phr; however, it decreases significantly when the NR dosage is 40 phr (a decrease of 16.5%), indicating that NR and CIIR cannot be effectively co-vulcanized under this condition [37]. The tensile strength of the vulcanizate first increases and then decreases with an increase in the dosage of EPDM in the system, thereby achieving a 15.6% increase when the EPDM dosages are 60 and 80 phr. This suggests that EPDM and CIIR in the rubber compound can be co-vulcanized effectively, resulting in improved mechanical properties.

The stress at a given elongation (SE) and hardness are both important indicators of stiffness, representing the force required to produce a certain deformation. SE is associated with larger tensile deformations, while hardness is measured with smaller compressive deformations [38]. The SE of a vulcanizate depends on the structure of the polymer molecular chain, vulcanization system, and reinforcing and filling systems. The larger the molecular weight of the rubber macromolecule, the fewer free ends it contains, which can lead to a larger SE. The interaction between the polymer macromolecules strengthens when the molecular chain contains polar groups or atoms, improving the deformation resistance of the vulcanizate and ultimately increasing its SE. The SE of the vulcanizate is affected by the type of crosslinking bonds and shows a linear relationship with the crosslinking density.

**Figure 6:**
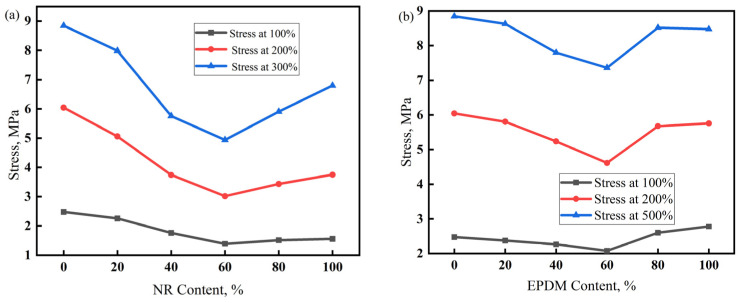
Stress at a given elongation (SE) of NR/CIIR vulcanized rubber (**a**) and EPDM/CIIR vulcanized rubber (**b**).

Figure 6 shows that, with an increase in the NR dosage in the NR/CIIR vulcanizate, its 100% SE first decreases and then increases. The 100% SE is 2.48 MPa for NR0, which decreases to 1.39 MPa when the NR dosage reaches 60 phr (a decrease of 43%). The 200% SE of the vulcanizate exhibits the same trend. NR0 has the largest 200% SE of 6.04 MPa, while NR60 has the lowest 200% SE of 3.02 MPa (a decrease of 50%). Similarly to the 100% and 200% SE, the 300% SE also shows a trend of first decreasing and then increasing. The 300% SE of NR60 is 4.94 MPa, which decreases by 44% from 8.85 MPa for NR0. Thus, the 100%, 200%, and 300% SE values of NR/CIIR vulcanizate all decrease with an increase in NR dosage, with the minimum SE being ~50% lower than the maximum value. The smallest SE is found when the NR dosage reaches 60 phr. This can be attributed to the formation of a two-phase continuous structure as the volume fractions of the NR phase, and the CIIR phase gradually become identical with an increase in NR dosage. In addition, the co-vulcanization of NR and CIIR is weak in NR60 without forming crosslinking structures between the two phases.

The SE of the EPDM/CIIR vulcanizate decreases first and subsequently increases with the increasing EPDM dosage, with EPDM60 causing the smallest SE. Unlike the SE changing trend of NR/CIIR, EPDM100 exhibits a higher SE value than that of EPDM0 at 100% strain, whereas EPDM0 shows a slightly higher SE value at 300% strain. Under smaller strains, the filler network starts to destruct in the vulcanizate, and the destruction dominates under larger strains. The above phenomena indicate that the EPDM phase bears more stress at smaller strains, whereas the CIIR phase bears more stress at larger strains. Similarly, the primary reason for the lowest SE in EPDM60 is the absence of a well-developed crosslinking network between the two phases.

### 3.3. Dynamic Mechanical Properties of EPDM/CIIR Blends and NR/CIIR Blends

From a damping mechanism perspective, internal friction refers to the capacity of macromolecular chains to dissipate mechanical energy under the action of an external force [39]. The magnitude of the internal friction depends on the mobility of the moving units in the macromolecule chain, including backbone bonds, side groups, chain links, and chain segments. Further, low internal friction can cause the macromolecular chain to freely orient and deform along the direction of the force.

**Figure 7:**
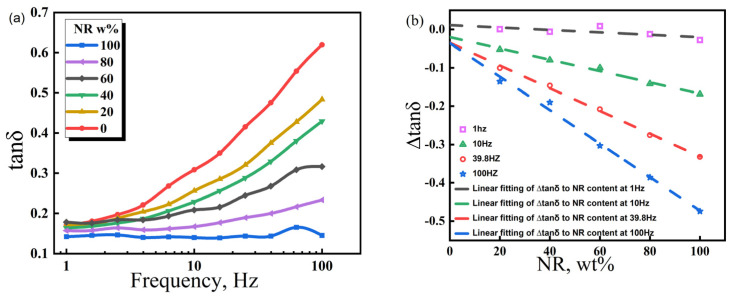
Variation law of the energy loss factor (*tanδ*) of NR/CIIR vulcanized rubber with scanning frequency (**a**) and difference in the energy loss factor (∆*tanδ*) between NR/CIIR vulcanized rubber and CIIR (**b**).

Figure 7 indicates that, at 20 °C, NR100 has the smallest loss factor (*tanδ*) in the frequency range of 1–100 Hz (~0.1), suggesting a poor damping performance. The *tanδ* of NR0 is high and increases with frequency in the range of 1–100 Hz. This demonstrates that periodic external forces at low frequencies are insufficient to trigger the CIIR molecular chain for overcoming frictional resistance and achieving relative movement. Only high-frequency periodic external forces can compel individual moving units to consume mechanical energy through the movement. Based on the molecular chain structure of the polymer, the densely arranged side methyl groups on the CIIR macromolecular skeleton have produced strong internal friction under the action of periodic external forces. During the transition from the low frequency to the high frequency, the NR/CIIR blend exhibits a *tanδ* plateau. The frequency range of the *tanδ* plateau indicates the frequency sensitivity of internal friction. The larger the range, the lower the frequency sensitivity. As shown in the figure, the *tanδ* plateau frequency range is the smallest for NR0 and the largest for NR100. The expanded range of the *tanδ* plateau at a high NR content is related to the good flexibility and low internal friction of the NR macromolecular chain. Therefore, the *tanδ* plateau frequency range is determined by the NR content. The higher the NR content, the lower the sensitivity of the NR/CIIR vulcanizates to changes in frequency.

Comparatively, NR/CIIR compounds have different internal frictions at the same frequency. A high NR content leads to a small *tanδ* [40]. The *tanδ* value of pure CIIR (referred to as NR0) is established as the standard to quantify the effect of NR content on the damping properties of NR/CIIR vulcanizates, and the *tanδ* of NR/CIIR vulcanizates with different NR contents is subtracted from the *tanδ* of NR0 for calculating ∆*tanδ.* The correlation between NR content and ∆*tanδ* of NR/CIIR vulcanizates at different frequencies can then be obtained.

∆tanδ=−0.000235973x+0.00538

∆tanδ=−0.00147x−0.02007

∆tanδ=−0.00321x−0.01654

∆tanδ=−0.00462x−0.01708

The above equations are the linear fits of ∆*tanδ* and NR content at 1, 10, 39.8, and 100 Hz, respectively. The intercept refers to the *tanδ* difference between the NR/CIIR vulcanizate with 0 phr NR and pure CIIR at a certain frequency. In principle, the intercept is supposed to be zero; however, because of experimental errors, this value slightly deviates from zero. A positive slope indicates that the addition of NR increases the *tanδ* of the rubber compound, which corresponds to improved damping performance at the given experimental temperature and frequency. When the slope is negative, it suggests that the addition of NR reduces the *tanδ* of the NR/CIIR blend, thereby resulting in a deterioration of the damping performance of the material under the experimental conditions. The absolute value of the slope indicates the damping performance of the NR/CIIR blend at different frequencies. A larger value shows the greater influence of NR dosage on the material damping performance at this frequency.

When the frequency is 1 Hz, the ∆*tanδ* value remains unchanged when the NR content varies from 0 to 100%. This implies that the damping performance of NR/CIIR vulcanizates is extremely insensitive to changes in the NR/CIIR ratio at an external force frequency of 1 Hz. The absolute slope obtained from the fitting becomes larger with an increase in the external force frequency. When the frequency is 100 Hz, ∆*tanδ* changes significantly when the NR content changes from 0 to 100%, indicating that the damping performance of the material is most sensitive to the NR dosage at 100 Hz. Therefore, plotting the variation in ∆*tanδ* with respect to NR content in a broader frequency range can assist in selecting the optimal ratio of raw rubber based on specific usage conditions, achieving the objective of cost reduction.

**Figure 8:**
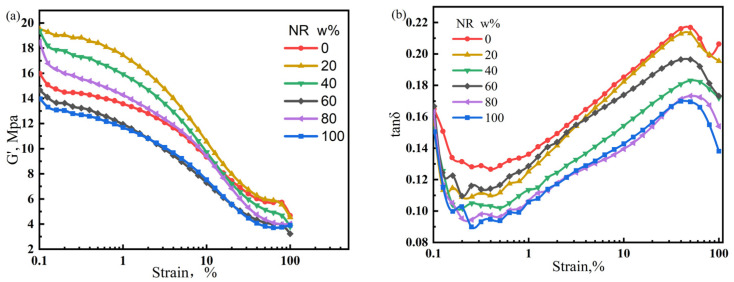
Variation in the storage modulus (**a**) and loss factor of NR/CIIR vulcanized rubber (**b**) with strain size.

The performance of rubber products is determined by the properties of the matrix and dispersion state of the filler. Poorly dispersed fillers can cause severe agglomeration, resulting in premature failure. In contrast, a uniform and stable filler dispersion can enhance the performance of rubber products. In this section, the Payne effect is used to characterize the dispersion of fillers in the NR/CIIR matrix. The storage modulus variation (ΔG’) of the NR/CIIR vulcanizates is shown in Figure 8.

The ΔG’ (difference between G’ values at 0% and 15% strain) value is used to quantify the significance of the Payne effect, and the calculated results are shown in Table 3.

The ΔG’ values of pure CIIR and NR are relatively small (7.89 MPa and 7.75 MPa, respectively). Blending NR with CIIR leads to an increase in ΔG’, implying a stronger Payne effect. The Payne effect is most significant when the NR dosage is 40 phr, with a ΔG’ of 11.37 MPa. When the NR dosage is 60 phr, the ΔG’ decreases to 8.55 MPa. The above results show that the Payne effect of NR/CIIR vulcanizate becomes more significant after blending.

The damping performance of the material is significantly affected by the moving units of the polymer macromolecule chain when the temperature, strain amplitude, and frequency of external force are identical. EPDM is a rubber copolymerized by ethylene, propylene, and a third monomer. Each propylene chain segment contains a side methyl group favorable for energy dissipation; however, EPDM only exhibits good damping performance at low temperatures because of its relatively narrow damping temperature range, good flexibility of the molecular chain, and low glass transition temperature. This section examined the EPDM dosage effect on the damping performance of EPDM/CIIR vulcanizates at frequencies of 1–100Hz, providing data support for further research on the damping performance of EPDM/CIIR vulcanizates.

**Figure 9:**
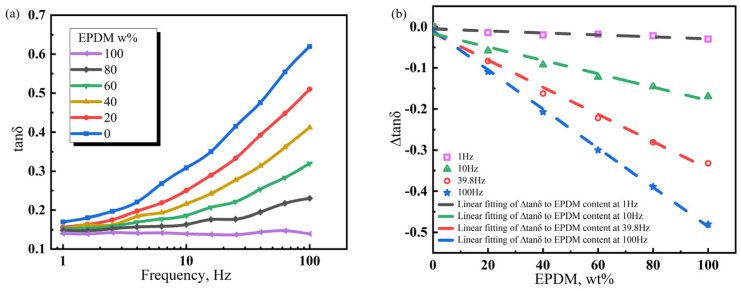
Variation law of the energy loss factor (*tanδ*) of EPDM/CIIR vulcanized rubber with scanning frequency (**a**) and difference in the energy loss factor (∆*tanδ*) between EPDM/CIIR vulcanized rubber and CIIR (**b**).

As shown in Figure 9, Within the tested frequency range, the *tanδ* of EPDM is stable, which slightly fluctuates around 0.15, indicating that the damping performance of EPDM vulcanizate is very insensitive to frequency changes in the range of 1–100 Hz. The frequency range of *tanδ* plateau narrows gradually with a decrease in the EPDM dosage. When EPDM80 is subjected to an external force frequency higher than 10 Hz, *tanδ* gradually increases, thereby showing an ~90% shrinkage in the *tanδ* plateau frequency range compared with that of EPDM100. When EPDM60 is stimulated by an external force frequency greater than 2.5 Hz, *tanδ* starts to increase, and its *tanδ* plateau frequency range is reduced by 97.5% compared with that of EPDM100. Four frequencies of 1, 10, 39.8, and 100 Hz were selected to quantify the EPDM dosage effect on the damping performance of EPDM/CIIR vulcanizates. The *tanδ* of EPDM0 at each frequency was used as a reference, and the *tanδ* of EPDM/CIIR vulcanizates with different EPDM dosages at the same frequency was subtracted from the *tanδ* of EPDM0 to obtain ∆*tanδ*. The obtained data points were fitted, and the quantitative relationships between ∆*tanδ* and EPDM dosage are expressed as follows:

∆tanδ=−0.0002414x−0.00497

∆tanδ=−0.00163x−0.01664

∆tanδ=−0.00331x−0.01491

∆tanδ=−0.00476x−0.00962

The above equations represent the relationships of ∆*tanδ* as a function of EPDM dosage at 1, 10, 39.8, and 100 Hz, respectively. The intercept refers to the difference in *tanδ* between the EPDM/CIIR vulcanizate with 0 phr EPDM and pure CIIR at a certain frequency, which should be zero. The smaller the deviation of the intercept from zero, the closer it is to the theoretical value. The slope represents the damping performance of EPDM/CIIR vulcanizates at different frequencies. Comparing the fitting results of EPDM/CIIR and NR/CIIR vulcanizates at various frequencies, their slopes are very similar. The slope exhibits the smallest change (2.3%) at a frequency of 1 Hz and the largest change (10.9%) when the frequency is 10 Hz. The above data show that when EPDM and NR are blended with CIIR, their influence on the damping performance of CIIR is essentially the same within the test frequency range. Therefore, NR and EPDM can replace each other when factors such as aging do not need to be considered in the formulation design and the damping properties of the vulcanizates are not changed. The EPDM dosage in EPDM/CIIR vulcanizates is linearly correlated with ∆*tanδ*. Therefore, the quantity of EPDM can be directly calculated according to the performance requirements when designing the formula of EPDM/CIIR vulcanizates.

**Figure 10:**
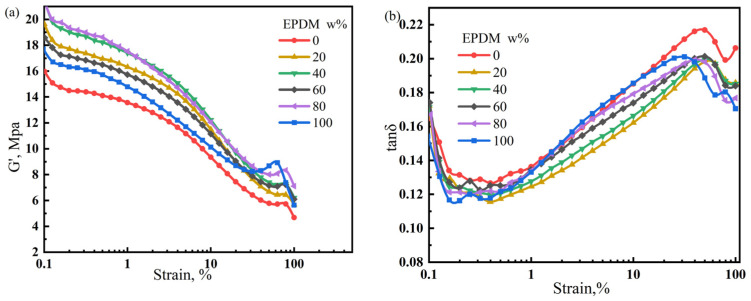
Variation in the storage modulus (**a**) and loss factor of EPDM/CIIR vulcanized rubber (**b**) with strain size.

For the EPDM/CIIR vulcanizates studied in this section, the magnitude of the Payne effect is determined by the EPDM dosage. The storage modulus variation (ΔG’) of the EPDM/CIIR vulcanizates is shown in Figure 10.

As shown in Table 4, Introducing EPDM increases the Payne effect of the vulcanizate. The ΔG’ value of EPDM40 is the highest (11 MPa), which is 39.4% higher than that of EPDM0. Except for EPDM0 and EPDM100, the Payne effect in EPDM60 is the smallest.

## 4. Conclusions

This study employed NR and EPDM to blend with CIIR. Their effects on the vulcanization characteristics, mechanical properties, and damping performance were investigated. As the NR component in the matrix increases, the scorch time of the mixed rubber increases first and then decreases. When the NR dosage is 20 phr, the scorch time of the mixed rubber is the longest, indicating that NR-20 has the best processing safety. When 20 parts of NR were used together, the t_90_ decreased by 40.2%, and the CRI increased by 132%. This indicates that the use of partial NR in CIIR can greatly improve the vulcanization speed of the mixed rubber and increase production efficiency. As the EPDM component in the matrix increases, the scorch time of the mixed rubber increases, and when the EPDM dosage exceeds 40 phr, the scorch time of the mixed rubber increases significantly. As the EPDM component in the matrix increases, the vulcanization rate of the rubber compound slows down. When combined with 20 phr EPDM, t_90_ increased by 24.9%, and CRI decreased by 20.6%. When the dosage of NR in NR/CIIR vulcanizate increases, the tensile strength of the vulcanizate first increases and then decreases before increasing again. Furthermore, when the NR dosage is 20 phr, the tensile strength of the NR/CIIR vulcanizate decreases by 16.5%. With increasing EPDM dosage in the EPDM/CIIR vulcanizate, the tensile strength first increases and then decreases. The tensile strength of EPDM/CIIR vulcanizates increases by 15.6% when the EPDM dosage is 60 and 80 phr. The damping performance of NR/CIIR vulcanizates at different frequencies was characterized. The fittings of ∆*tanδ* as a function of NR dosages at 1, 10, 39.8, and 100 Hz were performed for NR/CIIR. The slope represents the sensitivity of frequency to the NR dosage in vulcanizates, and a larger slope indicates a higher sensitivity. Dynamic Mechanical Properties tests on NR/CIIR vulcanizates demonstrate that the blending of NR and CIIR can enhance the Payne effect. The damping performance of EPDM/CIIR vulcanizates at different frequencies was characterized by using the same way to process the experimental data of the NR/CIIR vulcanizates. Comparing the fitting results of EPDM/CIIR and NR/CIIR vulcanizates at various frequencies revealed that their slopes are very similar. This implies that when EPDM and NR are blended with CIIR within the test frequency range, their effect on the damping performance of CIIR is essentially the same. Therefore, when the damping properties of the vulcanizates are not changed, NR and EPDM can replace each other. The effect of NR or EPDM dosages on NR/CIIR or EPDM/CIIR vulcanizates can be evaluated by fitting the data of ∆*tanδ* versus NR or EPDM dosage. Therefore, the quantity of NR or EPDM can be conveniently calculated based on the performance requirements when designing the formula. At present, there are few studies on NR\CIIR and EPDM\CIIR compound rubber. The research results involved in the article have important reference significance for relevant practitioners in rubber selection.

## Figures and Tables

**Table 1:** Formula composition of the NR/CIIR blend rubber material.

Component (phr)	NR0	NR20	NR40	NR60	NR80	NR100
CIIR	100	80	60	40	20	
NR		20	40	60	80	100
N550	50	50	50	50	50	50
Paraffin oil	5	5	5	5	5	5
Zinc oxide	5	5	5	5	5	5
Stearic acid	2	2	2	2	2	2
4020	1	1	1	1	1	1
DM	1	1	1	1	1	1
Sulfur	2	2	2	2	2	2

**Table 2:** Formula composition of the EPDM/CIIR blend rubber material.

Component (phr)	EPDM0	EPDM20	EPDM40	EPDM60	EPDM80	EPDM100
CIIR	100	80	60	40	20	
EPDM		20	40	60	80	100
N550	50	50	50	50	50	50
Paraffin oil	5	5	5	5	5	5
Zinc oxide	5	5	5	5	5	5
Stearic acid	2	2	2	2	2	2
4020	1	1	1	1	1	1
DM	1	1	1	1	1	1
Sulfur	2	2	2	2	2	2

**Table 3:** Storage modules of NR/CIIR vulcanized rubber.

	NR0 (CIIR)	NR20	NR40	NR60	NR80	NR100
G’_0_/MPa	15.9	19.5	19.3	14.6	18.5	13.9
G’_15_/MPa	8.0	8.7	7.9	6.1	7.6	6.2
G’_0_–G’_15_/MPa	7.8	10.7	11.3	8.5	10.8	7.7

**Table 4:** Storage modulus of EPDM/CIIR vulcanized rubber.

	EPDM0 (CIIR)	EPDM20	EPDM40	EPDM60	EPDM80	EPDM100
G’_0_/MPa	15.97	19.67	21.53	18.59	21.189	17.71
G’_15_/MPa	8.08	9.80	10.52	9.71	10.54	9.13
G’_0_–G’_15_/MPa	7.89	9.84	11.00	8.86	10.63	8.57

## Data Availability

The original contributions presented in this study are included in the article. Further inquiries can be directed to the corresponding author.

## Abbreviations

The following abbreviations are used in this manuscript:

CIIR	Chlorinated butyl rubber
NR	Natural rubber
EPDM	Ethylene propylene diene monomer rubber
IIR	Butyl rubber
TAC	Triallyl cyanate
MMA	Methyl methacrylate
MES	EPDM-g-MMA St/MS resin blend
CCA	Citric acid
MAH	Maleic anhydride
